# SARS-CoV-2 Infection: A Forerunner or Precursor in Anti-neutrophil Cytoplasmic Antibody-Associated Vasculitis With Kidney Injury

**DOI:** 10.7759/cureus.28705

**Published:** 2022-09-02

**Authors:** Zaw Thu Aung, Rotimi Oluyombo, Mahzuz Karim, Jessica Wong Sun Wai, Shiva Ugni

**Affiliations:** 1 Internal Medicine, Norfolk and Norwich University Hospitals, Norwich, GBR; 2 Renal Medicine, Norfolk and Norwich University Hospital, Norwich, GBR; 3 Norwich Medical School, University of East Anglia, Norwich, GBR; 4 Internal Medicine, Norfolk and Norwich University Hospital, Norwich, GBR; 5 Nephrology, Queen Elizabeth Hospital NHS Foundation Trust, Kings Lynn, GBR

**Keywords:** proteinuria, microscopic haematuria, : acute kidney injury, covid 19, small vessel vasculitis

## Abstract

COVID-19 disease and anti-neutrophil cytoplasmic antibody (ANCA)-associated vasculitis are both multi-systemic conditions. It is postulated there is a causal relationship between both conditions and this is supported by some case reports. The symptoms of COVID-19 can mimic those of vasculitis especially when the respiratory system is affected. Early diagnosis and treatment of ANCA-vasculitis cannot be overemphasized as this reduces the risk of severe organ damage. We report a 64-year-old lady with SARS-CoV-2 infection who developed ANCA-vasculitis with acute kidney injury and we reviewed the literature on this plausible association.

We performed an electronic search of the MEDLINE, EMBASE, CINAHL, and EMCARE databases for research studies and case series and reports published in the English language between April 2020 and February 2022. Our review suggests that patients with COVID-19 infection who had proteinase 3-ANCA positive vasculitis with diffuse alveolar haemorrhage had fatal outcomes. We also noticed an increased incidence of active urine sediments. We emphasize the importance of a high index of suspicion for diagnosis and early treatment of vasculitis to ensure an improved outcome.

## Introduction

COVID-19 disease, due to infection with severe acute respiratory syndrome coronavirus-2 (SARS-CoV-2), is a multisystem inflammatory disorder that can lead to endothelial damage, increased thrombo-embolic risk, cytokine storm, and autoimmune phenomena. Anti-neutrophil cytoplasmic antibody (ANCA)-associated vasculitis (AAV) is a systemic inflammatory autoimmune disease, predominantly affecting small vessels, and resulting in varied clinical manifestations depending on the organs involved. AAV is most commonly idiopathic but can be associated with other triggers such as infections or drugs [1]. It has been postulated that there is a similar causal relationship between COVID-19 and AAV, supported by a number of case reports [2]. Here, we describe a patient with PR3-positive AAV following SARS-CoV-2 infection and present a review of the literature.

Literature search strategy

We performed an electronic search of the MEDLINE, EMBASE, CINAHL, and EMCARE databases for research studies and case series and reports published in the English language between April 2020 and February 2022 with the following search terms: “Severe Acute Respiratory System Coronavirus-2” OR “SARS-CoV-2” OR “COVID-19” AND “Anti-Neutrophil Cytoplasmic Antibody-Associated vasculitis” OR “ANCA-vasculitis” OR “vasculitis”. We also included relevant studies or reports cited in the publications yielded by the search. The final list was checked for duplicate and non-relevant publications.

## Case presentation

A 64-year-old British woman with a background medical history of type 2 diabetes mellitus, essential hypertension, and asthma contracted COVID-19, which was confirmed with a positive PCR in February 2021. The symptoms of body ache, headache and lethargy were mild and she managed this herself at home, not requiring hospital admission.

Two months later, she presented to the hospital complaining of shortness of breath. She was afebrile, did not require supplemental oxygen (oxygen saturation 96%-98% on air) and was haemodynamically stable with a blood pressure of 130/70mmHg. Blood tests are shown in Table 1.

**Table 1:** Biochemistry and selected blood results of the patient ND - Not Done, CRP - C-reactive protein, PR3-ANCA - Proteinase 3-Antineutrophil Cytoplasmic Antibodies, MPO - Myeloperoxidase, Anti-dsDNA - anti-double-stranded deoxyribonucleic acid, GBM abs - glomerular basement membrane antibodies, C3 - complement 3, C4 - complement 4.

Parameters (units)	1^st^ presentation	2^nd^ presentation(3 weeks later)	Following treatment (9 months after 2nd presentation)	Reference range
Serum creatinine (µmol/L)	48	237	93	45-84
Urea (mmol/L)	6.2	15.3	6.8	2.5-7.8
Bicarbonate(mmol/L)	ND	24.1	ND	19-28
D-Dimer (ng/ml)	1183	1474	ND	<500
CRP (mg/L)	193	341	6	0-10
Haemoglobin (g/L)	120	80	112	130-170
White cell count (x10^9 ^cells/L)	8.1	8.13	7.2	4-10
Lymphocyte count (x10^9 ^cells/L)	0.6	0.6	0.5	1-3
Platelets (x10^9 ^cells/L)	260	528	300	150-400
PR3-ANCA(IU/mL)	ND	119	8.3	0-3
MPO-ANCA(IU/mL)	ND	0.2	0.1	0-5
ANA	ND	0.7	ND	0-1
Anti-dsDNA (IU/mL)	ND	9	ND	<10
Anti-GBM abs(U/mL)	ND	0	ND	0-10
C3 (g/L)	ND	0.9	ND	0.75-1.65
C4 (g/L)	ND	0.2	ND	0.14-0.54
Albumin creatinine ratio (mg/mmol)	ND	97.5	14	0-20

There were right middle zone changes on the chest radiograph. A diagnosis was made of community-acquired pneumonia, and she was discharged home with oral antibiotics. She re-presented to the hospital three weeks later with worsening dyspnoea together with haemoptysis, reduced urine output and visible haematuria. Her blood pressure was 142/69 mmHg, heart rate 100 bpm, and respiratory rate 22 per minute. Her oxygen saturations were low at 88%-90% on air, rising to 97% on high-flow oxygen. Heart sounds were normal, venous pressure was not elevated, and abdominal examination was unremarkable. Chest auscultation revealed widespread crackles with bronchial breath sounds and wheeze. Her arterial blood gas performed on oxygen administered at 15 L/min via a non-rebreathe mask revealed pH 7.43, pO_2_ 8.26 kPa, pCO_2_ 4.94 kPa, lactate 1.6 mmol/L and bicarbonate 24.1 mmol/L. Chest radiograph showed bilateral patchy changes.

**Figure 1:**
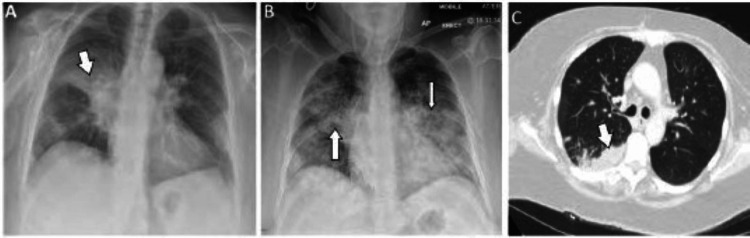
(A) Chest radiograph antero-posterior (AP) projection, showing right mid-zone heterogeneous opacity. (B) Chest radiograph AP projection showing diffuse bilateral heterogeneous opacities (white arrows). (C) Computed tomography scan of chest showing ill-defined patchy consolidation more prominent on the right side.

She proceeded to undergo a chest CT scan, which revealed ill-defined patchy consolidation consistent with a wide range of possible causes including infection, malignancy, or vasculitis. Figures 1A-1C show the radiological images taken during the initial and subsequent presentations.

She was transferred to the intensive care unit where she required endotracheal intubation and ventilation and was commenced on continuous veno-venous haemofiltration. COVID-19 PCR swabs, blood cultures, and atypical pneumonia screens (including Legionella and Pneumococcus) were negative. However, PR3 ANCA was elevated but with normal anti-nuclear antibodies and complements (C3 and C4) (Table 1). A diagnosis was made of AAV and she was given high dose intravenous steroids before transfer to a tertiary centre where she received further steroids (total of three doses of pulsed Methyl prednisolone 500 mg followed by prednisolone wean), intravenous cyclophosphamide (two pulses), and seven cycles of plasma exchange. She then received rituximab as a maintenance immunosuppression. She made a good clinical improvement and was subsequently discharged. At her latest follow up nine months after her initial hospital admission, she has received her third dose of the COVID-19 vaccine, and has remained well with improved immunology and other blood parameters (Table 1). She only mentioned occasional fatigue.

## Discussion

Acute kidney injury (AKI) occurs as a complication in around 20% of patients with COVID-19 and is associated with an increased risk of progression to end-stage renal disease (hazard ratio, HR, 2.96) [3,4]. An association between COVID-19 and crescentic glomerulonephritis (GN) has also been identified but is rare [5]. In addition, there is growing evidence of an association between COVID-19 and AAV, but the exact mechanism is not understood.

Excessive inflammatory responses and immune dysregulation play a major role in the pathophysiology of COVID-19. This can include increased activity of neutrophil extracellular traps (NETs) [6,7] and abnormal eosinophil function with excessive degranulation [8]. There can be an associated disruption of immunological self-tolerance, and indeed some studies have shown that up to half of patients with severe COVID-19 have auto-antibodies associated with conditions such as idiopathic thrombocytopenic purpura, Miller Fisher syndrome, Guillain-Barre syndrome, and Kawasaki disease [2]. In a study of 124 patients with COVID-19 (108 hospitalised, 16 asymptomatic), ANCA, anti-proteinase 3 (PR3), and anti-myeloperoxidase (MPO) levels were higher than in controls [9]. ANCA levels increased with the severity of the infection and fell as the disease waned. Interestingly, however, no patients had clinical features of AAV other than pulmonary abnormalities.

Vascular injury in AAV is mediated by priming and activation of neutrophils that can occur through some mechanisms including infection, drugs, or activation of the alternative complement pathway [10]. The activated neutrophils express antigens on their surfaces that can interact with ANCA. The rise in serum ANCA titres observed in patients with COVID-19 suggests that this may act as a neutrophil priming event [9]. 

The fact that both AAV and COVID-19 may affect the lungs and kidneys can pose a diagnostic challenge and lead to a delay in diagnosis, potentially worsening patient and renal outcomes. It is particularly important to identify patients who may have developed AAV following COVID-19. The two conditions have similar radiological chest findings, albeit, with subtle differences: for example, both can lead to ground glass opacities, but peripheral and lower zone involvement tend to predominate in COVID-19 [11]. Other features that may suggest the development of AAV in patients with recent COVID-19 include haemoptysis or the presence of cavitations, nodules, or pulmonary masses on thoracic imaging [12,13].

The majority (76%) of patients in our literature review (19 out of 25) had active urine sediments with haematuria and proteinuria while AKI was present in 44% compared with 19% in the COVID-19-only cohort [14]. Interestingly Morita et al. found that, while the activity of urine sediments increased with the severity of COVID-19 disease, this activity was lower than in patients with non-COVID-19-related AKI, matched for level of renal dysfunction [15]. This suggests that the presence of very active urinary sediment in patients with COVID-19 should prompt clinicians to consider other causes of kidney injury and have a low threshold for further investigation such as ANCA testing. This is of vital importance as outcomes in these patients are markedly improved by early diagnosis and the institution of appropriate therapy such as immunosuppression and plasma exchange.

Among the 25 cases, we found in the literature (median age of 49 years with 13 females) (Table 2), AAV was diagnosed one to six months after COVID-19 in 12 patients [11,16-26] while COVID-19 and AAV were diagnosed during the same admission in 11 cases [5,27-36].

**Table 2:** Characteristics and outcomes of cases reported in the literature ANA - antinuclear antibodies; ANCA - antineutrophil cytoplasmic antibodies; DAH - diffuse alveolar haemorrhage; dsDNA - double-stranded DNA; ECMO - extracorporeal membrane oxygenation; GBM - glomerular basement membrane; HD - haemodialysis; HSP - Henoch Schoenlein purpura; IVIg - intravenous immunoglobulin; MPO - myeloperoxidase; NA - not available; PEx - plasma exchange; PMH - past medical history; PR3 - Proteinase 3; RF - Rheumatoid factor; RNP - ribonuclear protein, NM - Not Mentioned

References	Age (years)	Gender	Background Medical History	Immunology	Biopsy	AKI at diagnosis	Interval to ANCA diagnosis	Urine findings at diagnosis of vasculitis	Presence of DAH	Treatment	Outcome
Uppal et al., 2020 [5]	64	M	Cryptogenic organising pneumonia	ANA, RNP, Anti-dsDNA, p-ANCA (MPO)	Kidney	Yes	Same admission	Haematuria and proteinuria	NM	Prednisolone, rituximab, HD	Survived
	46	M	Diabetes	c-ANCA (PR3)	Kidney	Yes	Same admission	Haematuria and proteinuria	NM	Methylprednisolone, rituximab	Survived
Manivannan et al., 2021 [11]	41	F	Obesity, chronic sinusitis	c-ANCA (PR3)	No	No	1 month	NA	Present	IV steroid, cyclophosphamide, PEx, ECMO	Died
Asma et al., 2022 [16]	72	M	NA	c-ANCA (PR3)	Kidney	Yes	2 months	Haematuria and proteinuria	NM	Corticosteroid, cyclophosphamide	Survived
Garcia-Vega et al., 2022 [17]	60	M	Hypertension	p-ANCA (MPO)	Kidney	Yes	3 months	Haematuria and proteinuria	NM	Methylprednisolone, rituximab	Survived
Wang et al., 2021 [18]	56	F	Asthma	ANA, p-ANCA (MPO)	No	No	2 months	NA	Present	IV steroid, cyclophosphamide	Survived
Fireizen et al.,2021 [19]	17	M	Obesity, asthma	p-ANCA (MPO)	Kidney	No	2 months	Haematuria and proteinuria	Present	Steroid, cyclophosphamide, PEx	Survived
Izci et al., 2021 [20]	26	M	None	ANA, p-ANCA (MPO)	Kidney	No	NA	Haematuria and proteinuria	Present	Methylprednisolone, cyclophosphamide, HD, PEx	Survived
Patel et al., 2021 [21]	51	M	None	c-ANCA (PR3), low C3 and C4	No (coagulopathic)	No	1 month	Haematuria and proteinuria	Present	HD	Died
Morris et al., 2021 [22]	53	M	NA	c-ANCA (PR3), low C3 and C4	No (coagulopathic)	No	1 month	Haematuria and proteinuria	Present	Methylprednisolone, HD	Died
Jalalzadeh et al., 2021 [23]	46	F	Diabetes mellitus, scleroderma	ANA, Anti-RNP, p-ANCA (MPO)	Kidney	No	6 months	Haematuria and non-nephrotic range proteinuria	Present	Methylprednisolone, rituximab	NA
Allena et al., 2021 [24]	60	F	Hypertrophic obstructive cardiomyopathy, coronary artery disease, asthma, hypertension, hyperlipidaemia	ANA, p-ANCA (MPO)	Kidney	No	1 month	Haematuria and nephrotic range proteinuria	Present	Methylprednisolone, rituximab	Survived
Selvaraj et al., 2021 [25]	60	F	Diabetes mellitus, allergic rhinitis	c-ANCA (PR3)	Kidney	No	1 month	Haematuria and proteinuria	Present	Methylprednisolone, rituximab	Survived
Wali et al., 2021 [26]	26	F	No known PMH	p-ANCA, anti-GBM	Kidney	No	1 month	Haematuria and proteinuria	Present	Methylprednisolone, rituximab	Survived
Zakrocka et al., 2021 [27]	59	M	Hypertension	p-ANCA	No	Yes	Same admission	Proteinuria and haematuria	NM	Methylprednisolone, cyclophosphamide, HD, PEx	Died
Wintler et al., 2021 [28]	13	F	HSP	c-ANCA (PR3), low C4	Yes	NA	Same admission	Proteinuria and Haematuria	NM	Methylprednisolone, rituximab	Survived
Reiff et al., 2021 [29]	17	M	None	c-ANCA (PR3)	Lung	No	Same admission	NA	NM	Methylprednisolone, rituximab	Survived
Cobilinschi et al., 2021 [30]	67	F	Hypertension, dyslipidaemia	ANA, RF p-ANCA (MPO	No	Yes	Same admission	Haematuria and proteinuria	NM	Corticosteroid, cyclophosphamide	Survived
	36	F	None	c-ANCA (PR3)	Kidney	No	Few weeks	Haematuria and proteinuria	NM	Steroid, cyclophosphamide	Survived
Martati et al., 2021 [31]	64	F	Hypertension	c-ANCA (PR3), anti-cardiolipin, anti-β2 glycoprotein-I IgM	Kidney	Yes	Same admission	Haematuria and proteinuria	NM	Steroid, PEx,HD, cyclophosphamide (then switched to rituximab as developed anti-phospholipid syndrome)	Survived
Powell et al., 2021 [32]	12	F	Hypertension	p-ANCA (MPO)	Kidney	NA	Same admission	Haematuria and proteinuria	Present	Methylprednisolone, rituximab, cyclophopsphamide	Survived
Chargui et al., 2021 [33]	49	M	NA	p-ANCA (MPO)	Kidney	Yes	Same admission	Haematuria and proteinuria	Present	Methylprednisolone, cyclophosphamide, PEx	Died
Fares et al., 2020 [34]	55	F	Stroke, hypertension, asthma	ANA, p-ANCA (MPO)	No	Yes	Same admission	NA	Present	Methylprednisolone, PEx	Died
Hussein et al., 2020 [35]	37	F	No previous PMH	c-ANCA (PR3)	No	Yes	Same admission	Red cell casts	Present	Methylprednisolone, PEx	Died
Moeinzadeh et al., 2020 [36]	25	M	No previous PMH	c-ANCA (PR3)	Kidney	Yes	Same admission	Proteinuria	Present	Methylprednisolone, PEx, IVIg, cyclophosphamide	Survived

**Figure 2:**
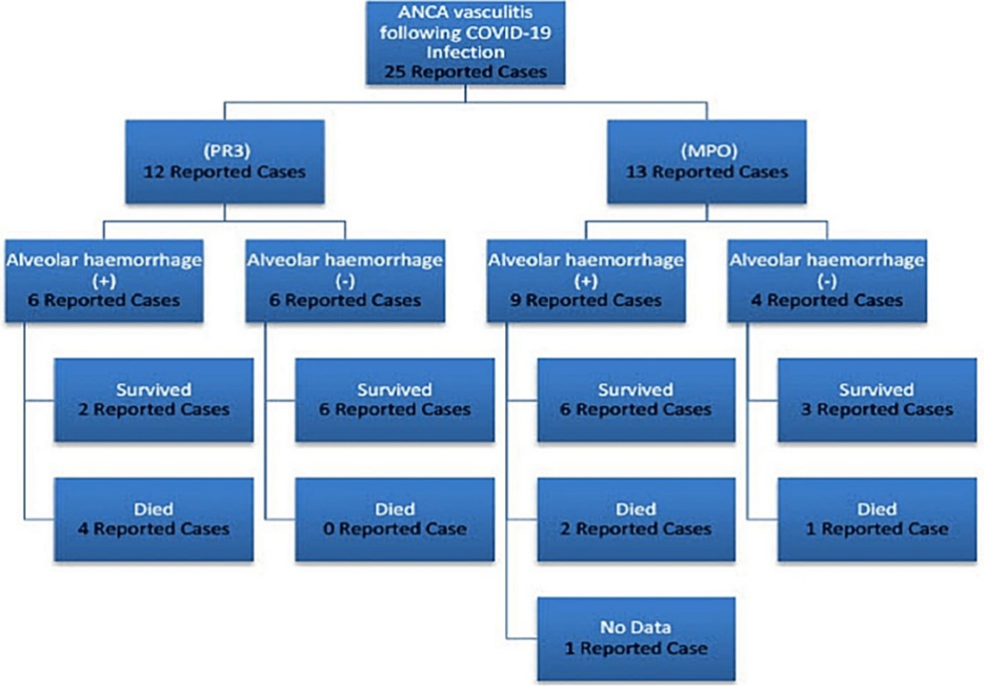
Summary of ANCA subtypes, presence of alveolar haemorrhage, and patients’ outcome ANCA - Anti-Neutrophil Cytoplasmic Antibody

It is noteworthy that severe AAV with life-threatening alveolar haemorrhage can follow a mild COVID-19 infection, exemplified by two patients who had mild COVID-19 (managed with self-quarantine but not requiring hospital admission) but subsequently died following pulmonary haemorrhage [12,21]. In general, pulmonary haemorrhage is seen in up to 40% of patients with AAV and confers an increased risk of mortality by nine times [37]. In this review six out of the seven patients who died had suffered an alveolar haemorrhage (Figure 2), emphasizing the importance of early diagnosis.

## Conclusions

In conclusion, SARS-CoV-2 infection may precipitate the development of vasculitis. A high index of suspicion is important in patients with recent or current COVID-19 where there are atypical clinical features such as haemoptysis, active urinary sediment, or atypical findings on chest imaging. This will facilitate early diagnosis and maximise the chance of optimising patient outcomes such as mortality, residual chronic kidney disease, or progression to end-stage renal disease. Further studies on the association between COVID-19 and AAV may yield a valuable further understanding of the pathophysiology of both conditions.
